# Are Lunatics Criminals?

**Published:** 1873-11

**Authors:** 


### *Are Lunatics Criminals?*

The subject of the legal status of criminal lunatics has absorbed the attention of the medical and legal professions and of the public so deeply of late, that there is some danger of confounding the greater with the lesser category, and regarding all who may be so unfortunate as to have lost their wits as criminals.

Whether this danger exist theoretically may or may not be true, but certainly the practical effects of such a theory are already apparent in this city; and while the sufferer from small-pox or a broken leg is tenderly conveyed to befitting hospital accommodation provided for his reception by the county commissioners, the still more unfortunate sufferer from disease of the brain is dragged like a felon to the nearest police station or to the county jail, to share the cells of vagabonds and convicts. It would be well if some of our philanthropists (and there are many true ones in our midst) could visit occasionally the County Court of Cook county upon a day when the victims of brain disease are brought before it for examination. They would then see, perhaps, some respected citizen, some valued friend, some beloved relative, even, who had been decided to be insane, sent, by order of the court, to the jail below. The learned and humane judge who presides over this court deplores most deeply the necessity which compels him, having due regard to the protection of society, to order the incarceration of these unfortunate beings in the only place at his disposal for the purpose.

This state of affairs is a crying evil, demanding the prompt attention of the county commissioners, to whose notice the subject has been brought repeatedly and urgently, but thus far without avail. Will they wait until one of their own number shall be reduced to this fearful condition, and, becoming too violent to be controlled by his family, be locked up in the filthy cells of this abode of horrors, to become the jest of malefactors, and the prey of vermin?

Within a few days a young gentleman, for many years a resident of the North Side, and a member of a well-known commercial firm, had an attack of epileptic mania, during which he became so violent as to endanger the safety of his young wife and infant child. In default of other asylum he was consigned to a cell of the nearest police station house, and, after examination before the County Court, to the county jail, until arrangements could be made for placing him in the asylum for the insane. Could the county commissioners have seen the grief of that broken-hearted young wife and mother, on parting from her husband at the threshold of his prison, their hearts, perhaps, would have been awakened to the necessity of providing a temporary asylum for such cases.

We have seen, in a cell of a police station house in this city, the wife of a respectable citizen, who by reason of her insanity could not be properly cared for at home, in a perfectly nude condition, exposed to the gaze of any policeman or curious idler who might choose to peer through the grating of her cell-door, the apartment itself being too filthy to justify its use even by an animal.

Our county commissioners have at last provided a receptacle for the unrecognized dead, the "Morgue," and we hope will some day provide for living sufferers from all disease whether of body or mind; until they do this, an essential part of their functions remains undischarged.

It is to be hoped that the people, through the secular press, their mouth-piece, will clamor without cessation until this urgent need is supplied. H.

